# Metabolite signatures of doxorubicin induced toxicity in human induced pluripotent stem cell-derived cardiomyocytes

**DOI:** 10.1007/s00726-017-2419-0

**Published:** 2017-04-18

**Authors:** Umesh Chaudhari, James K. Ellis, Vilas Wagh, Harshal Nemade, Jürgen Hescheler, Hector C. Keun, Agapios Sachinidis

**Affiliations:** 1 Institute of Neurophysiology and Center for Molecular Medicine Cologne (CMMC), University of Cologne (UKK), Robert-Koch-Str. 39, 50931 Cologne, Germany; 2 Division of Cancer, Department of Surgery and Cancer, Faculty of Medicine, Imperial College London, London, UK

**Keywords:** ^1^H NMR metabolomics, Cardiotoxicity, Toxicity prediction, Metabolite biomarkers, Pluripotent stem cells, Cardiomyocytes

## Abstract

Drug-induced off-target cardiotoxicity, particularly following anti-cancer therapy, is a major concern in new drug discovery and development. To ensure patient safety and efficient pharmaceutical drug development, there is an urgent need to develop more predictive cell model systems and distinct toxicity signatures. In this study, we applied our previously proposed repeated exposure toxicity methodology and performed ^1^H NMR spectroscopy-based extracellular metabolic profiling in culture medium of human induced pluripotent stem cell-derived cardiomyocytes (hiPSC-CMs) exposed to doxorubicin (DOX), an anti-cancer agent. Single exposure to DOX did not show alteration in the basal level of extracellular metabolites while repeated exposure to DOX caused reduction in the utilization of pyruvate and acetate, and accumulation of formate compared to control culture medium. During drug washout, only pyruvate showed reversible effect and restored its utilization by hiPSC-CMs. On the other hand, formate and acetate showed irreversible effect in response to DOX exposure. DOX repeated exposure increased release of lactate dehydrogenase (LDH) in culture medium suggesting cytotoxicity events, while declined ATP levels in hiPSC-CMs. Our data suggests DOX perturbed mitochondrial metabolism in hiPSC-CMs. Pyruvate, acetate and formate can be used as metabolite signatures of DOX induced cardiotoxicity. Moreover, the hiPSC-CMs model system coupled with metabolomics technology offers a novel and powerful approach to strengthen cardiac safety assessment during new drug discovery and development.

## Introduction

Drug-induced cardiotoxicity is a leading cause of drug attrition in drug discovery and development. Hence cardiac safety assessment remains a critical component in preclinical drug development. In addition to electrophysiological studies, multiple parameters need to be considered to evaluate series of toxic events of pharmaceutical compounds. Currently used safety assessment parameters are informative but not sufficient to exclude the potential cardiotoxic effects of new drugs for human. Moreover, till now the experimental in vitro and in vivo animal models cannot truly predict cardiotoxicity in humans because of physiological differences between different species. Such model systems can identify false negative compounds, which can cause serious structural and functional damage to the human heart. Therefore pharmaceutical industries urgently need novel and diverse biomarkers, and also clinically relevant in vitro experimental models. Toxicological studies routinely use biomarkers to evaluate harmful effects of compounds. Advancement in ‘-omics’ technologies like transcriptomics and proteomics has accelerated the process of new biomarker discovery. Metabolomics is an emerging ‘-omics’ technology that offer new parameter of global metabolic profiling and is a promising tool for biomarker discovery (Johnson et al. 2016; Lewis et al. 2008). Nuclear Magnetic Resonance (NMR) and Mass Spectroscopy (MS) are routinely used in the metabolic profiling of biological samples. Compared to MS, NMR is less sensitive but highly reproducible, with other advantages over MS (Markley et al. 2016). NMR spectroscopy-based metabolomics can quantify low-molecular weight metabolites in biological samples (cells/tissues/organism), biological fluids (serum/urine) and cell culture media. NMR-based metabolite profiling has been used to detect drug responsive to early changes in metabolite levels contributing to the subsequent toxicity (Andreadou et al. 2009; Park et al. 2009; Zhang et al. 2015). Such toxicity-responsive metabolite signatures can be used to predict adverse effect of drug candidates at an early stage of preclinical drug development (Clarke and Haselden 2008; Robertson et al. 2011; Wishart 2008).

Human induced pluripotent stem cell-derived cardiomyocytes (hiPSC-CMs) beat spontaneously, recapitulate human cardiac biology and show clinically relevant physiological response upon exposure to drugs (Liang et al. 2013; van Meer et al. 2016). Since hiPSC-CMs may overcome the limitations of existing experimental models used in cardiac safety assessment, they are becoming reliable source for high-throughput drug discovery screening, toxicity prediction, cardiac disease modeling, regenerative medicine and for wide range of life sciences studies (Chow et al. 2013; Ebert et al. 2012; Sharma et al. 2013).

Doxorubicin (DOX) is a highly effective chemotherapeutic drug used in the treatment of various types of cancer including solid tumors, hematological malignancies and soft tissue sarcoma. However, its chemotherapeutic use is mainly limited by the occurrence of dose-dependent cardiotoxicity (Gianni et al. 2008; Zhang et al. 2012). The DOX induced cardiotoxicity is manifested by acute cardiac dysfunction, chronic cardiomyopathy and progressively leading finally to congestive heart failure. Although extensive work has been done on DOX, the mechanism of DOX mediated cardiotoxicity is still not completely understood. Different types of molecular mechanisms are proposed including oxidative stress, topoisomerase II beta inhibition and alteration in mitochondrial energetics (Minotti et al. 2004; Octavia et al. 2012; Zhang et al. 2012). Cellular energetics plays a critical role in DOX induced cardiomyopathy (Tokarska-Schlattner et al. 2006).

In this study, ^1^H NMR spectroscopy-based metabolite profiling was performed in extracellular culture medium of hiPSC-CMs exposed to DOX or solvent control. The metabolic profiling was performed to identify early metabolic signatures in response to DOX exposure. The present findings from metabolomics measurements are consistent with DOX-induced disturbances in mitochondrial metabolism. Our study demonstrates that metabolic profiling of media from hiPSC-CM cultures in the presence of cardiotoxicants can provide a unique platform to predict the cardiotoxic potential of pharmaceutical drug candidates.

## Materials and methods

### Cardiomyocytes cell culture

Highly pure population (>98%) of human induced pluripotent stem cell-derived cardiomyocytes (iCell Cardiomyocytes^®^, Cellular Dynamics International, Madison, WI, USA) were used to perform all the experiments. These spontaneously beating cardiomyocytes are mixture of atrial, ventricular and nodal like myocytes. For metabolomics studies, cryopreserved hiPSC-CMs were thawed as per manufactured instructions and resuspended in iCell cardiomyocytes plating medium (iCell-PM; Cellular Dynamics International, Madison, WI, USA) and plated on fibronectin (5 µg/cm^2^, 2 h at 37 °C) (Sigma-Aldrich, Steinheim, Germany) coated 6-well cell culture grade plate at a density of approximately 0.4 × 10^6^ cells/well. Two days post plating; hiPSC-CMs were refreshed with and maintained in iCell-cardiomyocytes maintenance medium (iCell-MM; Cellular Dynamics International, Madison, Wi, USA) with fresh media change after every 2 days. Four days post-plating hiPSC-CMs were used for compound exposure. The cardiomyocytes were cultured in standard cell culture incubator under culture conditions of 5% CO_2_ and 37 °C.

### Reference compound

10 mM stock solution of Doxorubicin (Sigma-Aldrich, Steinheim, Germany) was prepared in high quality dimethyl sulfoxide (DMSO) solvent. Stock solution was dispensed into small volume aliquots and stored at −20 °C for long-term use. iCell-MM was brought to room temperature prior to drug dilutions.

### Experimental design and compound exposure

**Fig. 1:**
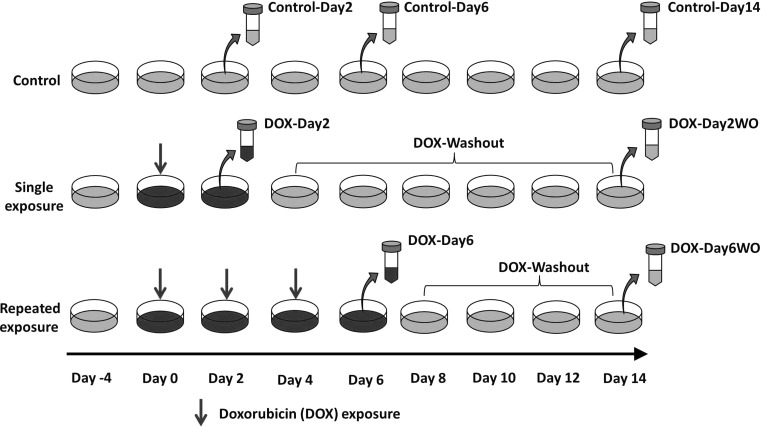
A schematic view of experimental design with timeline of the study. Synchronously beating monolayer of hiPSC-CMs was exposed to DOX for 2 days or for 6 days in a repeated way (DOX-supplemented culture media was refreshed every 2 days). Following 2 and 6 days exposure to DOX, hiPSC-CMs were further cultured in drug free culture medium until day 14 (after the start of drug exposure). Culture media was refreshed every 2 days. For metabolic profiling, culture media samples were collected on day 2, 6 and 14. Experimental design was also used in cytotoxicity and ATP content determination studies

For metabolic profiling studies, we used our previously established in vitro repeated exposure toxicity methodology (Fig. 1) (Chaudhari et al. 2016). In short, hiPSC-CMs were given single exposure to DOX (156 nM) for 2 days (DOX-Day2) or three consecutive repeated exposures to DOX (DOX-Day6) (DOX supplemented media refreshed every 2 days). Following DOX exposure (single/repeated), hiPSC-CMs were cultured in drug free iCell-MM till day 14 after the start of drug exposure (DOX-Day2WO and DOX-Day6WO). Control-Day2 and -Day6 cardiomyocytes were exposed to DMSO as a solvent control. Culture medium was refreshed every 2 days.

### Sample collection and metabolomics analysis

Metabolomics samples were collected from three independent experiments. As shown in Fig. 1 the experimental media samples were collected from the cardiomyocytes culture medium both during drug exposure (Day 2 and Day 6) and drug washout (Day 14 after start of first drug exposure). Media samples from control cardiomyocytes (vehicle control) were also harvested at Day 0, 2, 6 and 14 time points. Media samples were collected in sterile tubes and immediately stored at −80 °C. Culture media samples represent the metabolite changes in the spent culture media over a culture period of the previous 2 days. Media that had not been exposed to cells was analysed to determine the initial concentration of individual metabolites, which allowed us to determine if the change in concentration observed was a result of cellular influx or efflux. For metabolomics studies, the media samples were thawed on ice. A DSA (4,4-dimethyl-4-silapentane-1-ammonium trifluoroacetate [D_2_O (50 µL)] solution was added to the media sample (550 µL) to give a final DSA concentration of 0.97 mmol/L. The samples were centrifuged to remove any particulate matter and transferred to a 5 mm NMR tube (Norell, USA). 1D Carr–Purcell–Meiboom–Gill (CPMG) NMR spectroscopy and data processing was carried out as previously described (Ellis et al. 2011; Keun and Athersuch 2011).

### Determination of ATP content and LDH release

High energy ATP molecules functions as a biomarker for metabolically active cells, and lactate dehydrogenase (LDH) release is general cytotoxicity biomarker. The iPSC-CMs were plated in fibronectin coated white 96-well plate at a density of approximately 20 × 10^3^ cells/well using iCell-PM. After 2 days of culture, iCell-PM was refreshed with iCell-MM. Four days post-plating, cardiomyocytes were exposed to DOX (156 nM) as per experimental design shown in Fig. 1. On day 2, 6 and 14, cardiomyocytes were used for ATP content determination, while culture media was used to measure enzymatic activity of LDH, released by cardiomyocytes in response to DOX exposure.

ATP amount was measured using ATPlite Luminescence ATP detection assay system kit (Perkin Elmer, Netherlands), according to the manufacturer’s instructions. In short, viable cardiomyocytes in the plate were lysed with mammalian cell lysis solution for 5 min in an orbital shaker. Lysed cells were immediately incubated with substrate solution for 5 min on an orbital shaker and then kept it undisturbed for 10 min in the dark place. After incubation, luminescence was measured on Softmax Pro M5e 96-well plate reader (Molecular Devices, Sunnyvale, CA, USA). Background luminescence was also measured from blank wells containing culture medium without cardiomyocytes. Emitted luminescence values are directly proportional to the amount of ATP.

Extracellular LDH activity was assessed using the Thermo Scientific^™^ Pierce^™^ LDH Cytotoxicity Assay Kit (colorimetric method) according to the manufacturer’s instructions. In brief, each media sample was gently mixed with reaction mixture, followed by incubation in the dark for 30 min at room temperature. The absorbance was measured at 490 nm using Softmax Pro M5e 96-well plate reader. Increase in absorbance is directly proportional to the LDH activity in the culture media. LDH activity in the culture medium without cardiomyocytes was considered as baseline activity.

## Results

### Analysis of the metabolomics data

**Fig. 2:**
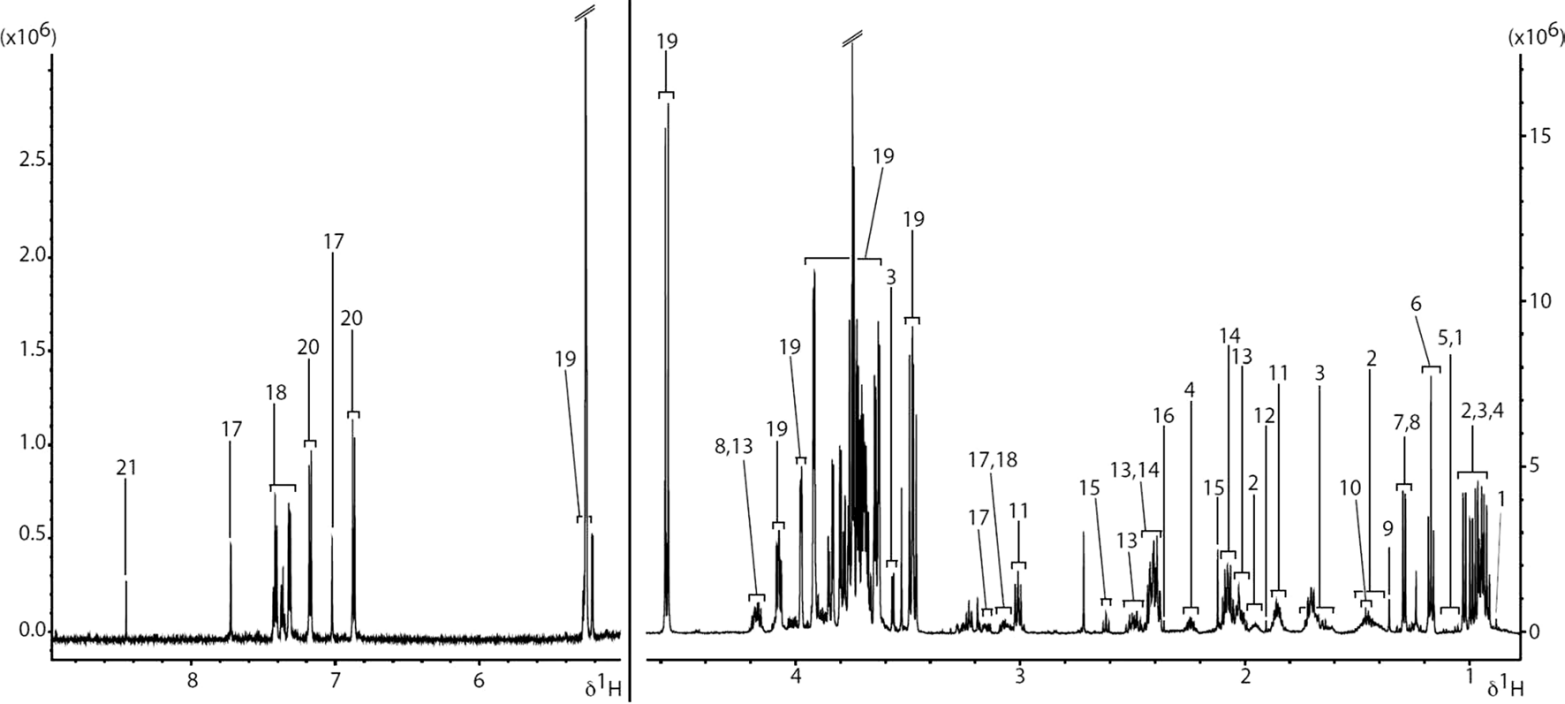
An extracellular metabolic profile of hiPSC-CMs model. The regions of the spectrum shown in the figure are a typical full resolution ^1^D 600 MHz ^1^H CPMG spin-echo NMR spectrum from the control media samples after 2 days of incubation. *1* 3-methyl-2-oxovalerate, *2* isoleucine, *3* Leucine, *4* valine, *5* 3-hydroxyisobutyrate, *6* ethanol, *7* lactate, *8* threonine, *9* 2-hydroxyisobutyrate, *10* alanine, *11* lysine, *12* acetate, *13* pyroglutamate, *14* glutamine, *15* methionine, *16* pyruvate, *17* T-methylhistidine, *18* phenylalanine, *19* galactose, *20* tyrosine, *21* formate

**Fig. 3:**
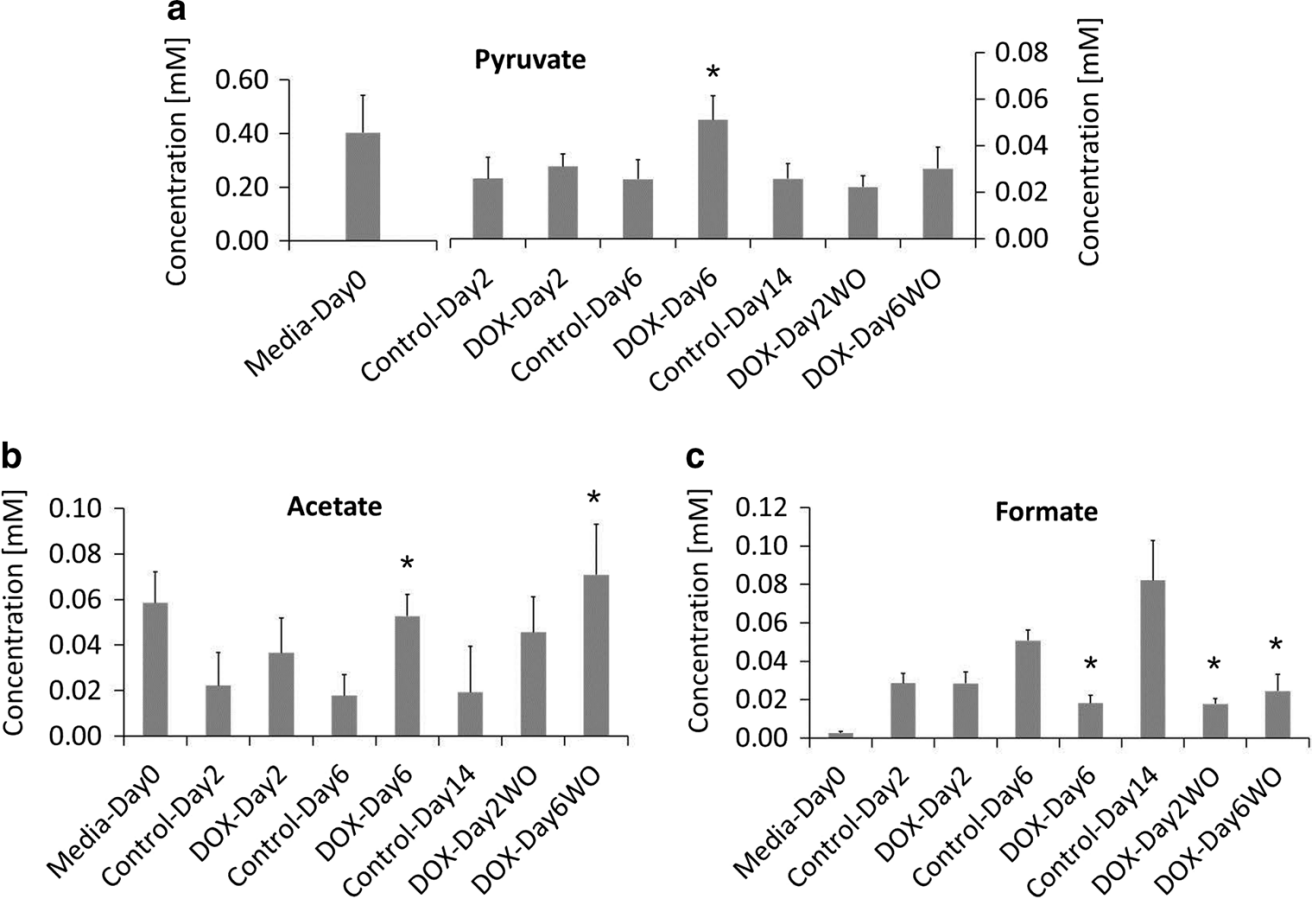
NMR spectroscopy detected significant alterations in extracellular metabolites levels of hiPSC-CMs. DOX reduced uptake of **a** pyruvate, **b** acetate, and **c** reduced efflux of formate. During drug washout pyruvate showed a reversible effect, while acetate and formate showed a irreversible effect. *Error bars* in the *bar* graphs represents standard deviation (SD). *n* = 3 biological replicates, *p value 0.05

For metabolic profiling studies, we used our previously established in vitro repeated exposure toxicity methodology (Fig. 1) (Chaudhari et al. 2016). ^1^H NMR spectroscopy was applied in metabolic profiling of culture medium of hiPSC-CMs model. A representative ^1^H CPMG spectrum of the spent culture media (Control-Day2) is shown in Fig. 2. Specific resonances are assigned to metabolites where possible and a full list of assigned spectral regions is listed in Table 1 with chemical shift ranges indicated. The chemical shift, in the units of ppm, describes where in the NMR spectrum a specific metabolite resonance is detected. In addition to the characterization of the extracellular metabolites at baseline, we investigated the influx and efflux of individual metabolites in culture media of hiPSC-CMs during and after DOX exposure (drug washout). Control media samples were used to determine changes in metabolite profile of DOX exposed media samples. No significant difference was observed between metabolic profile of Control-Day2 and DOX-Day2 groups. However, media samples from DOX-Day6 exhibited distinct changes in the metabolic profile compared to Control-Day6. Specifically, of the 20 metabolites studied, 3 showed a significant DOX-related effect in at least one treatment group; formate, pyruvate and acetate (Fig. 3). Either reversible or irreversible DOX-induced changes in the levels of these three metabolites were detected by ^1^H NMR metabolomics.

**Table 1:** Metabolites observed by 1D ^1^H NMR spectroscopy in the extracellular cell culture media

Metabolite	Ppm range	
2-Hydroxyisobutyrate	1.353	1.368
3-Hydroxyisobutyrate	1.048	1.073
3-Methyl-2-oxovalerate	0.868	0.900
3-Methyl-2-oxovalerate	1.102	1.122
Acetate	1.904	1.914
Alanine	1.450	1.474
Ethanol	1.155	1.190
Ethanol	3.640	3.660
Formate	8.437	8.449
Glutamine/pyroglutamate	2.401	2.456
Isoleucine	0.909	0.944
Isoleucine	0.985	1.008
Isoleucine	1.932	1.990
Lactate	1.310	1.330
Leucine	0.934	0.964
Leucine	1.603	1.756
Lysine	1.816	1.904
Lysine	2.993	3.038
Methionine	2.120	2.130
Methionine	2.645	2.601
Phenylalanine	7.296	7.329
Phenylalanine	7.337	7.382
Phenylalanine	7.390	7.435
Pyroglutamate	1.999	2.050
Pyroglutamate	2.456	2.476
Pyroglutamate	2.476	2.537
Pyroglutamate	4.094	4.120
Pyruvate	2.357	2.368
T-methylhistidine	3.038	3.129
T-methylhistidine	3.130	3.187
T-methylhistidine	7.707	7.744
Threonine	1.280	1.318
Threonine	3.557	3.602
Tyrosine	6.843	6.895
Tyrosine	7.149	7.192
Valine	0.962	0.985
Valine	1.014	1.042
Valine	2.221	2.281

Assigned metabolites are listed alphabetically and the chemical shifts ranges (“ppm ranges”) shown for each resonance. The chemical shift (units: ppm) range describes the region of the NMR spectrum that was manually selected to integrate the area under the peak (AUP) for a specific metabolite resonance, i.e. the AUP was calculated for the single peak that corresponds to the acetate resonance from 1.904 to 1.914 ppm

DOX exposure showed a temporal effect on the uptake of only one media component; pyruvate. Unlike single exposure, media samples collected from DOX-Day6 group showed significant reduction in the utilization of pyruvate and acetate (Fig. 3a, b). During drug washout, a reversible effect was observed in the utilization of pyruvate, while acetate showed an irreversible effect with high concentration levels in the culture medium. Control media samples exhibited increasing concentration levels of formate (effluxed by hiPSC-CMs) in a culture condition dependent manner. Compared to Control-Day6, DOX exposure in DOX-Day6 group significantly inhibited the accumulation of formate in the culture media (Fig. 3c). In addition, formate was the only metabolite for which production was irreversibly impaired by DOX, as evidenced by a significantly reduced level of formate in DOX-Day2WO and DOX-Day6WO media samples after drug washout. These findings suggest that DOX impaired mitochondrial metabolism in hiPSC-CMs.

**Fig. 4:**
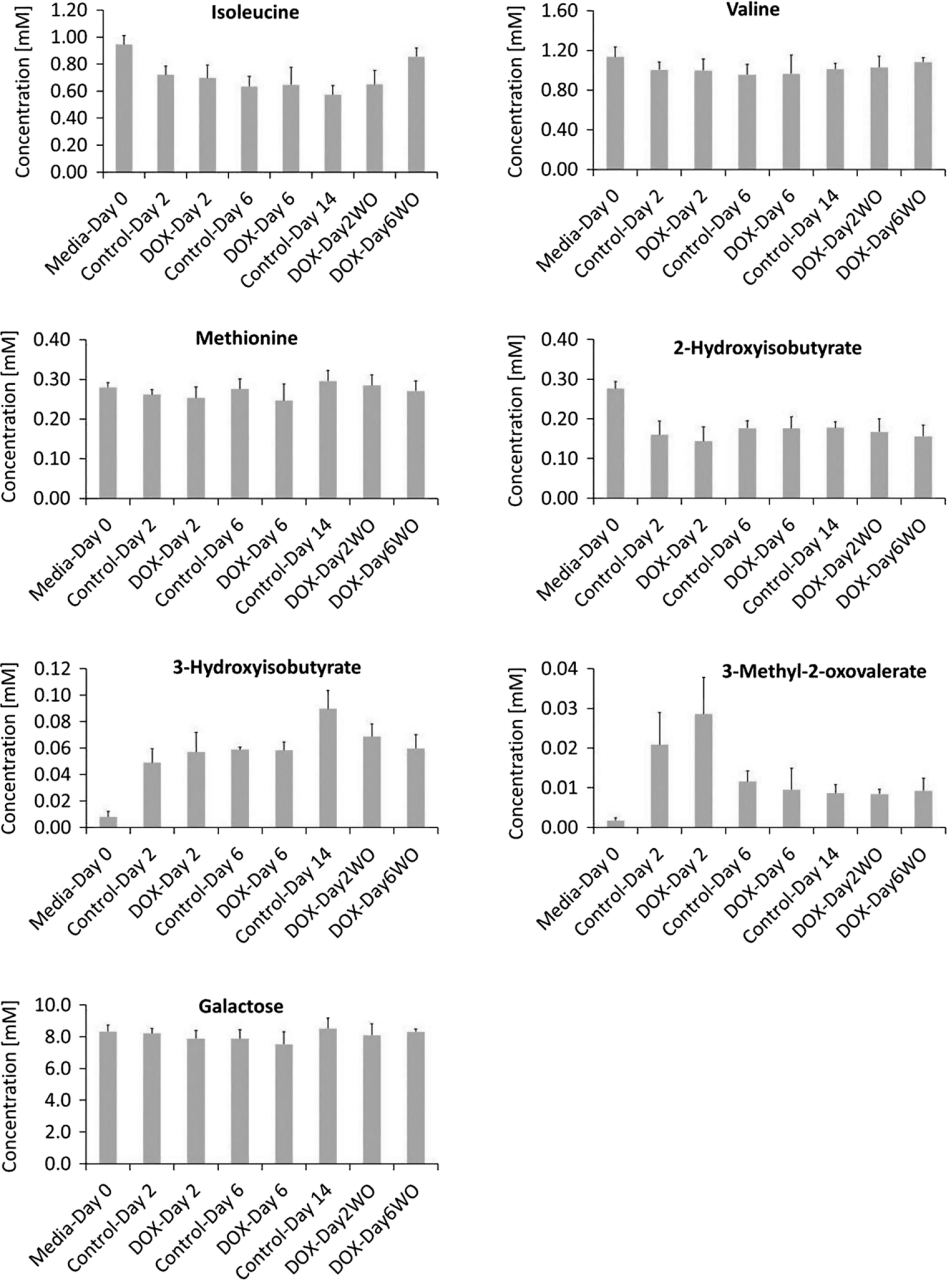
Response of extracellular metabolites- valine, 3-methyl-2-oxovalerate, isoleucine, tyrosine, 3-hydroxyisobutyrate, methionine and 2-hydroxybutyrate to DOX exposure in hiPSC-CMs model. ^1^H NMR spectroscopy was used to calculate the concentration (mM) of the metabolites in the culture media at day 2, 6 and 14 of DOX study. The levels of the metabolite are the net accumulation of influx and efflux over the previous 2 day period

No significant changes in the utilization of amino acids; valine, isoleucine and methionine (Fig. 4) could be observed. Leucine and lactate concentrations could not be calculated due to interference from other metabolites. In addition, no significant changes were found in the 3-methyl-2-oxovalerate, 2-Hydroxyisobutyrate and 3-hydroxyisobutyrate concentration levels (Fig. 4).

### DOX induced LDH leakage from hiPSC-CMs

**Fig. 5:**
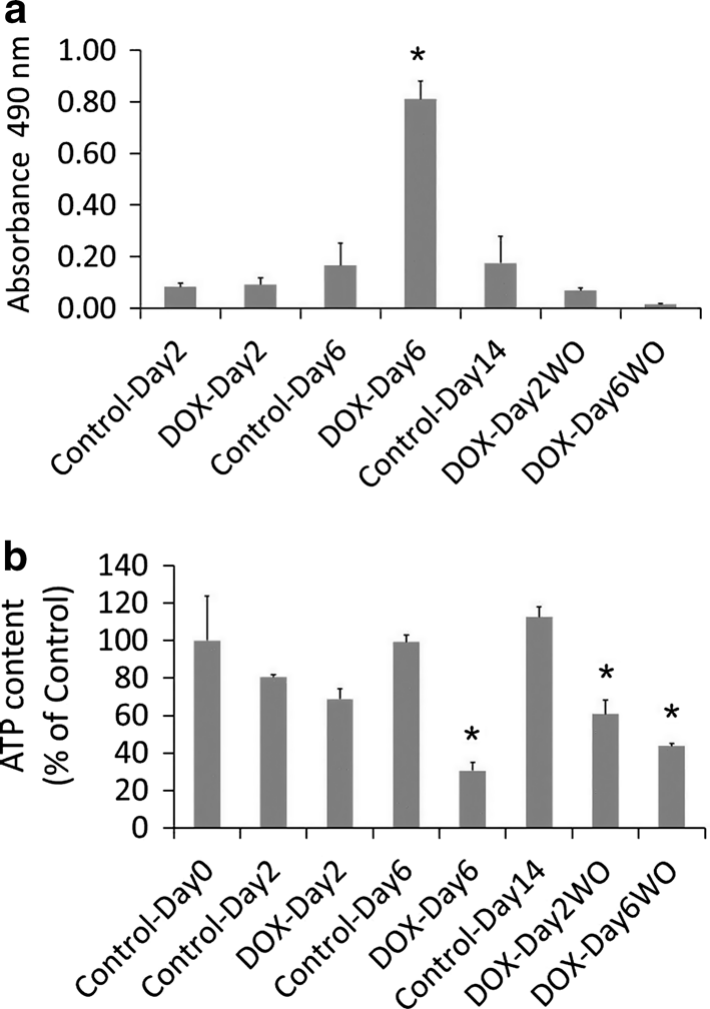
Determination of DOX induced cytotoxicity and energy levels in hiPSC-CMs. **a** DOX on repeated exposure induced cytotoxicity, as evidenced by increased LDH activity in the culture media. **b** DOX exposure declined energy level in cardiomyocytes with depletion of high energy ATP molecules. Data in *bar* graphs presented as mean ± SD. *n* = 3 technical replicates, *p value 0.05

LDH is impermeable to viable cell membrane, but it is released by cells in culture medium/blood stream in response to membrane damage and compound induced cytotoxicity. Compared to Control-Day2, media samples of DOX-Day2 did not show an increase in LDH activity (Fig. 5a). On the other hand, in media samples of DOX-Day6 group LDH release was observed with significant increase in LDH activity as compared to Control-Day6. Increased LDH activity indicates DOX induced cell membrane damage and cytotoxicity. Relative to Control-Day14, no significant increase in LDH activity was observed in drug washout media samples (DOX-Day2WO and DOX-Day6WO).

### DOX reduced ATP levels in hiPSC-CMs

ATP measurement was performed to determine cellular energy level after DOX exposure. Compared to controls, DOX exposure dependent depletion in the ATP levels was observed in DOX-Day2 and DOX-Day6 (Fig. 5b). Interestingly during drug washout, hiPSC-CMs from DOX-Day2WO group could not restore ATP levels, but a further decrease in ATP content was observed. Similarly hiPSC-CMs repeatedly exposed to DOX could not restore ATP levels after the removal of DOX (DOX-Day6WO). These data indicate a prolonged effect of DOX on depletion of the ATP-pool in hiPSC-CMs.

## Discussion

NMR-based metabolic profiling is a flexible approach to identify pharmaceutical compounds with potential physiological toxicity. Moreover, metabolomics platform may offer metabolites as toxicity biomarkers in preclinical predictive toxicity screening. For the first time we applied the NMR-based spectroscopy in metabolic profiling of hiPSC-CMs model system to detect extracellular metabolite signatures of DOX induced toxicity.

In this context, we applied our in vitro repeated exposure toxicity methodology (Chaudhari et al. 2016) to identify DOX response early, intermediate and long-term affected extracellular metabolites in hiPSC-CMs model system. Our previous cytotoxicity studies combined with functional studies based on impedance measurements demonstrated that DOX at a concentration of 156 nM was non-toxic, while its repeated exposure induced significant arrhythmicity and cytotoxicity to hiPSC-CMs (Chaudhari et al. 2016). These impedance based data were consistent with our LDH-based cytotoxicity data. The present metabolomics data suggests that hiPSC-CMs are non-responsive to a single exposure of DOX as assessed by extracellular metabolite levels. DOX responsive metabolic profiling of intracellular metabolites could be conducted in hiPSC-CMs to detect early toxicity metabolite signatures. On the other hand hiPSC-CMs induced distinct changes in the concentration level of certain extracellular metabolites in response to repeated exposure to DOX.

High energy demands of normal beating heart largely depend on a steady supply of pyruvate and fatty-acyl coenzyme A (Kolwicz et al. 2013). Acetate is also an important source of acetyl Coenzyme A (acetyl-CoA) and plays a significant role in maintaining energy homeostasis in mammalian cells (Shimazu et al. 2010). Acetyl-CoA is primarily utilized in anabolic processes such as fatty acid biosynthesis and an energy generating catabolic process; tricarboxylic acid (TCA) cycle (Gaspar et al. 2014). In the mitochondria, pyruvate and acetate derived acetyl-CoA, enters into the TCA cycle to meet energy requirements (Gaspar et al. 2014). In this study, decreased uptake of pyruvate and acetate from the culture media was observed after repeated exposure to DOX. This may reflect reduced ability of mitochondria to metabolize pyruvate and acetate to generate energy. Studies in rat-heart mitochondria indicated that the rate of pyruvate transport was inhibited by DOX (Paradies and Ruggiero 1988). However, during drug washout the reversible effect in the uptake of pyruvate suggests DOX dependent disturbances in the uptake/utilization of pyruvate. Interestingly, hiPSC-CMs repeatedly exposed to DOX exhibit irreversible effect in the uptake of acetate during drug washout which remained at high levels in culture media. In addition, hiPSC-CMs given single exposure to DOX showed reduction in the uptake of acetate, as evidenced by higher levels of acetate compared to controls (not statistically significant). Our data suggest that DOX induced disturbances in acetate relevant metabolic energy pathways. Acetate utilization and production could play important role in ageing process too (Shimazu et al. 2010). In this context, a previous in vivo study (Andreadou et al. 2009) utilized NMR spectroscopy to demonstrate significantly increased acetate (and succinate) levels in heart tissue exposed to DOX and the authors proposed these intracellular metabolites as a novel biomarker for DOX induced cardiotoxicity.

Formate is produced in the mitochondria by folate-mediated metabolic processes, and it is utilized in purine biosynthesis (Lamarre et al. 2013; Morrow et al. 2015). However, our knowledge about formate metabolism in the context of cardiac metabolism is limited. Formate, the only measured metabolite produced (effluxed) by hiPSC-CMs, was irreversibly affected during repeated exposure to DOX and after removal of DOX (drug washout). Our data gave evidence that DOX inhibits the folate-mediated mitochondrial production of formate. Therefore, changes in the formate metabolism may represent an early indicator of general mitochondrial damage. Reduced accumulation of formate during drug washout suggested a prolonged effect of DOX on the formate production and anabolic processes such as purine biosynthesis. However, the reduced production of formate by DOX may also occur due to an inhibition of one or more transporters responsible for the formate efflux. ATP molecules are mostly synthesized in the mitochondria and used as energy source in various cellular functions. DOX induced depletion in ATP levels was reported in rat and mice cardiomyocytes (Octavia et al. 2012; Pointon et al. 2010). Consistent with these observations, we demonstrated that DOX induced a decline in ATP levels in hiPSC-CMs. In general, the ATP depletion and mitochondrial dysfunction induced by DOX may be responsible for the DOX induced cardiotoxicity. In this context, impaired ATP production in mitochondria has been linked to cardiac hypertrophy and heart failure (Ingwall and Weiss 2004; Rosca et al. 2013).

## Conclusions

In summary, our study demonstrates that ^1^H NMR spectroscopy-based profiling of extracellular metabolites can be used to identify metabolic signatures associated with cardiotoxicity and cardiomyopathies. Pyruvate, acetate and formate can be proposed as candidate biomarkers to predict DOX induced physiological toxicity in the heart. Alterations in extracellular metabolite concentrations of these three metabolites may deepen our knowledge in understanding the mechanism of DOX induced cardiotoxicity. In general, the ATP depletion and mitochondrial dysfunction induced by DOX may be the main causes for the DOX induced cardiotoxicity. Our data suggest that metabolic profiling in hiPSC-CMs model system is an effective approach to strengthen preclinical cardiac safety assessment in new drug discovery and development.

## Abbreviations

Acetyl-CoA: Acetyl-coenzyme A
ATP: Adenosine triphosphate
CPMG: Carr–Purcell–Meiboom–Gill
DMSO: Dimethyl sulfoxide
DOX: Doxorubicin
DSA: 4,4-Dimethyl-4-silapentane-1-ammonium trifluoroacetate
hiPSC-CMs: Human induced pluripotent stem cell-derived cardiomyocytes
iCell-MM: iCell cardiomyocytes maintenance medium
iCell-PM: iCell cardiomyocytes plating medium
LDH: Lactate dehydrogenase
MS: Mass spectroscopy
NMR: Nuclear magnetic resonance
SD: Standard deviation
TCA cycle: Tricarboxylic acid cycle
